# Thirteenth Semi-Annual Meeting

**Published:** 1872-01

**Authors:** 


### Military Tract Medical Association — *Thirteenth Semi-Annual Meeting.*

Pursuant to adjournment, the Thirteenth Semi-Annual Meeting of this Association convened at Masonic Hall, in Galesburg, Tuesday, January 9th, at 10 o'clock, A. M., S. P. Breed, of Princeton, as President, Herbert Judd, of Galesburg, Secretary. After reading the record of the previous meeting, the Board of Censors reported several candidates for membership, who were elected Dr. Breed then called Dr. Reece to the chair, and presented the Association with a gavel, accompanying the act by the following very pleasant and appropriate remarks, which were kindly received by the members present:

*Mr. President, and Gentlemen of the Military Tract Medical Association:*

As a slight token of my esteem for the members of this body, and a sort of index of the deep interest I feel in the continued success of this Association, I beg leave to present to you, at this time, this *gavel.* While it will be at once apparent to all that this little instrument is a type of *authority* and *order,* it will be recognized only by the advanced student of physical science that it also represents, most appropriately, a certain measure of reserved or potential power, called statical energy. The organic structure of which the wood of this mallet is composed, may be said to represent just that amount of those physical forces, light, heat, electricity, etc., in a latent or statical condition, which will be restored to a sensible or dynamical condition by the resolution of this structure into its original inorganic elements by combustion or destructive metamorphosis, and thereby display the measure of its organic dynamics. These forces have been gradually stored up in this structure during the growth of the wood, from the surrounding media—the air and the earth—by the genial rays of the distant sun, to be given out again, on the oxidation or destruction thereof, in the form of light, heat, electricity, mechanical power, or some other correlative display of equivalent force, which may, or may not, be utilized in the arts or necessities of civilized life.

In this representation of the physical forces, however, this organic structure does not differ essentially from all other kinds of wood. But, in selecting the particular kinds of wood for this instrument of command, I have not been unmindful of those qualities which I thought would be appropriate to the occasion, and moreover, significant of the traits of character which I desire more particularly to elucidate.

The handle of this gavel is made of white cedar, or arbor vitæ. The propriety and fitness of this selection will at once appear, I hope, when it is remembered that, by a vertical section of the human cerebellum, just such a display of the white and gray matter is made, as to give, in miniature, a perfect picture of this beautiful tree, and is therefore named by all anatomists, "the arbor vitæ" in brain. Hence, gentlemen, you will now understand, if you have not done so before, that each one of us, unconsciously, perhaps, carries about constantly a perfect image or likeness of this "tree of life" engraven on our smaller brain. If this be so, it becomes doubly interesting to us, then, to know what are the peculiarities and most striking qualities of this tree.

It is an emblem of *persistence* and *fidelity;* two very important requisites for success in any business or profession. It is one of the finest *evergreens*, and is, in consequence, often selected as the ground-work for festoons, wreaths, and garlands, to deck the brow of the hero, the warrior, and the poet.

This tree is much employed by horticulturists and landscape gardeners for wind-brakes, screens, and ornamental hedges, and therefore may represent a shield—protection, and a higher order of *taste—refinement.*

In these qualities it is significant of the great mission of the true physician. Our first and greatest duty is to protect and shield mankind against the fell destroyer, disease. Indeed, it is the *privilege* and *duty* of the physician to so apply his knowledge, and so regulate his deportment, as to be, at the same time, a *protection* and *ornament* to society.

This tree is very tolerant of the pruner's shears. It may be trimmed, cut back, or trained to almost any shape. It may be restrained in *one* direction and stimulated in another, to almost any desired extent. Hence, it has become a type of *patience*, *docility*, and *tractability*. From the rude touch of the meddlesome it only returns an agreeable, aromatic fragrance; thus returning good for evil, and thereby illustrating the golden rule. So much for the handle.

The head of this gavel is made of apple wood. The most striking characteristics of this wood are, *firmness*, *solidity*, and *weight*, with a marked susceptibility of a high polish. It will not be denied that *firmness* of *purpose*, *solidity* of *reasoning*, and *weight* of *character* are *prime* elements to success for the physician.

This tree is remarkable for its *symmetry*, *thrift*, and *hardiness*—most excellent qualities, I assure you.

As a shade tree, it is scarcely inferior to any other; and, during its season of foliage, flowering, and fruitage, it is unsurpassed for its beauty of form, fragrance of flower, and abundance and value of its fruit. When we consider fully the value everywhere attached to the fruit, so grateful to the palate, so necessary to the health, so convenient and acceptable an offering to our guests, on almost all occasions, so diversified in its *role* in the culinary art, and for making cider and vinegar, we have an array of qualities unequaled by any other tree known to man; so that we even doubt, sometimes, whether the "Pomum Adami" is really the *apple* that our primeval mother Eve ate, and is still sticking in our throats.

But there is one other peculiarity about this tree and its fruit that I desire to call your attention to. It is the great improvement made in the quality of the fruit by judicious and appropriate cultivation. The parent of our common apple is admitted to be the wild crab of Europe. Would you know what can be done by patient labor in horticulture, persistently directed by the hand of science? Compare the little, gnarly, sour, wild crab, with some of our late varieties; such, for example, as the Bellflower, the Golden Pippin, and Wagoner, and you have the answer. This tree, then, above all others, is a type of progress. How appropriate and significant, then, is it to the live physician.

But last, though perhaps not the strangest of all, is the fact that this tree has its enemies—its parasites. In this respect, too, it is interesting and instructive to us. With all our personal efforts to benefit mankind, all our self-sacrificing devotion to relieve human suffering, we, too, have our *enemies*—our *parasites*. There are men among us in the community, who are so deaf that they never once hear the voice of sympathy, if it come from a physician. They are too blind to see the lenient hand as it brushes the tear of bitter anguish from the faded cheek, and too obtuse to properly realize that there is any virtue in the many sleepless nights, and days of toil, through wet and cold, to relieve the sick, the suffering and the needy.

Yes, gentlemen, there are such men found in every neighborhood, whose sensibilities are so gross and perverted, that they can see nothing praiseworthy in all this, and their influence is destructive of all good, and inimical to the most exalted virtues. They are like the vile *borer* in the heart of the good apple tree, and the coddling moth, and disgusting caterpillar, that riot among its flowers and prey upon its branches.

Gentlemen, we, too, like the good apple tree, have our parasites. Unfortunately there are men in every community who live, thrive and fatten on our very blood. They gather from our great storehouse of knowledge, but, ingrates as they are, they make no return. They even affect to contemn the very source of their success. They filch an *idea* here and a *fact* there, which enables them by artful dodges, cunning tricks and bold effrontery, to gain a temporary popularity with the ignorant, the credulous and the malcontent, whom they generally manage to fleece, spoil, and leave half dead, for the regular physician (the Good Samaritan) to pick up, comfort and care for as best he can.

These traveling mountebanks—these itinerant charlatans and nostrum venders—are like the pestiferous bark-lice which suck the juices, and greedily devour the pabulum intended to support the legitimate branches and tender twigs. These unscrupulous pretenders live, like other parasites, by a system of destruction, fraud and downright robbery. But their days are numbered. The skirmish-line of the great vanguard of progress, working patiently away, will, inevitably, *soon* drive them forever from the field. Then shall medicine see the dawn of a brighter and a better day. In view of this certain event, gentlemen, let us take courage, toil on and hope, for "surely the morning cometh."

If these few remarks shall serve, in any degree, to stimulate the weak, comfort the tried, and encourage the faltering, they will accomplish all I intended.

Dr. E. S. Phillips then read an essay, reviewing the history of medicine from an early period to the present time. This was an able and interesting production, and was accepted by the association with a vote of thanks.

*Reports from Standing Committees.* Dr. M. Reece, from the Committee on Surgery, reported a new method of treating strangulated hernia, referring to the practice of Drs. Cumming and Morton, of Louisville, Ky. This report was continued in detail. Dr. Reece favored the operation, which consists of simply puncturing the hernia tumor with a small canula needle, permitting its gaseous and fluid contents to escape, thus reducing the bulk and facilitating replacement.

Dr. Reece also placed on exhibition one of "Squire's vertebrated prostatic catheters," which he had had occasion to use with success. Dr. Hamilton, of Galesburg, spoke favorably of the lead catheter. Dr. Hamilton also presented, with history, the bones from a recent case, showing condition of knee joint from anchylosis of long standing. These reports were of much value to the association, and received due consideration.

*Materia Medica.* Dr. Webster, of Monmouth, read a paper, showing, in a clear and scientific manner, that this branch is in rapid progression, its study no longer that of a mass of incoherent precepts. To demonstrate the principles set forth in this paper, Quinia was chosen.

Dr. Marshall, of Monmouth, spoke of Digitalis in a way to elicit the views and experience of the members present. "Does it act as an arterial sedative by diminishing the force and frequency of the heart's contractions? or does it possess the opposite quality of stimulating and increasing the cardiac action?"

After this discussion morning session adjourned.

AFTERNOON SESSION.

Dr. C. A. Lambert, of Galesburg, upon *Ophthalmology*, read a paper on Trachoma; several cases were reported from his practice, with treatment and results. This report was the most scientific of any given, and received with benefit by the association.

*Obstetrics.* Dr. H. S. Hurd, of Galesburg, gave a statistical report from fifty cases, as occurring in his practice during the year past. This report was valuable, particularly to the younger members of the profession.

Upon the *Practice of Medicine* no reports were given from the standing committee. Interesting verbal reports were made, when the association was addressed by Dr. J. E. O'Brien, of Chicago.

His appeal in behalf of Rush Medical College met with worthy response. It was decided that the plan of scholarship was the most practicable, as but few members were present, and that a committee be appointed, composed of one member from each county belonging to the territory of the association. Said committee being appointed, was instructed to canvass among the members of the association, that one or more scholarships might be secured by the payment of $500 each. On motion, Knox county was allowed two members on said committee, which is as follows:

Dr. Wm. Hamilton, Galesburg, Knox county.
Dr. John M. Morse, " " "
Dr. G. W. Crossley, Princeton, Bureau county.
Dr. Hiram Nance, Kewanee, Henry county.
Dr. W. D. Craig, Aledo, Mercer county.
Dr. D. McMarshall, Hopper's Mills, Henderson county.
Dr. J. R. Webster, Monmouth, Warren county.

Under *Miscellaneous Business*, Dr. Aldrich presented a stomach from a late case that had been of doubtful diagnosis while under treatment, which was pronounced to have been chronic gastritis.

The Committee on Necrology were instructed to report at the next meeting on the deaths of Drs. Trimble, of Sagetown, and Chamberlain, of Princeton.

Eighteen delegates were elected to attend the State Medical Society in May.

Ten delegates were elected to attend the American Medical Association.

The following standing committees are to report at the next annual meeting in June:

*Censors*—J. R. Webster, S. M. Hamilton, Hiram Nance.

*Essayists*—A. V. T. Gilbert, J. R. Webster.

*Surgery*—M. Reece, S. K. Crawford, J. C. Smiley.

*Practice of Medicine*—Cephas Parks, David McDill, E. L. Phillips.

*Materia Medica*—Hugh Marshall, D. McMarshall, E. F. Purdum.

*Obstetrics, and Diseases of Women and Children* — J. W. Hensley, A. Klingberg, N. D. Sterling.

*Ophthalmology*—J. H Thompson.

Some forty members were present. This meeting was of interest and profit to all, being the most thoroughly practical of any meeting held by this association. The next meeting will be held on the second Tuesday in June, at Galesburg.

